# On a new law of bone remodeling based on damage elasticity: a thermodynamic approach

**DOI:** 10.1186/1742-4682-9-51

**Published:** 2012-11-29

**Authors:** Ahmed Idhammad, Abdelmounaïm Abdali

**Affiliations:** 1 Laboratory of Applied Mathematics and Computer Science (LAMAI), Faculty of Sciences and Technics, Abdelkrim El Khattabi Avenue, Marrakech, Morocco

**Keywords:** Numerical simulation, Thermodynamic approach, Small perturbations hypothesis, Bone remodeling, Bone density, Damage, Fatigue, Osteocyte, Elasticity, N-unit elements

## Abstract

**Background:**

Bone tissue is the main element of the human skeleton and is a dynamic tissue that is continuously renewed by bone-resorbing osteoclasts and bone-forming osteoblasts.

The bone is also capable of repairing itself and adapting its structure to changes in its load environment through the process of bone remodeling.

Therefore, this phenomenon has been gaining increasing interest in the last years and many laws have been developed in order to simulate this process.

**Results:**

In this paper, we develop a new law of bone remodeling in the context of damaged elastic by applying the thermodynamic approach in the case of small perturbations.

The model is solved numerically by a finite difference method in the one-dimensional bone structure of a n-unit elements model.

**Conclusion:**

In addition, several numerical simulations are presented that confirm the accuracy and effectiveness of the model.

## Introduction

Bone is a living material that constantly replaces old tissue with new in a process called remodeling. It is also able to respond adaptively to its environment [1,2].

**Figure 1:**
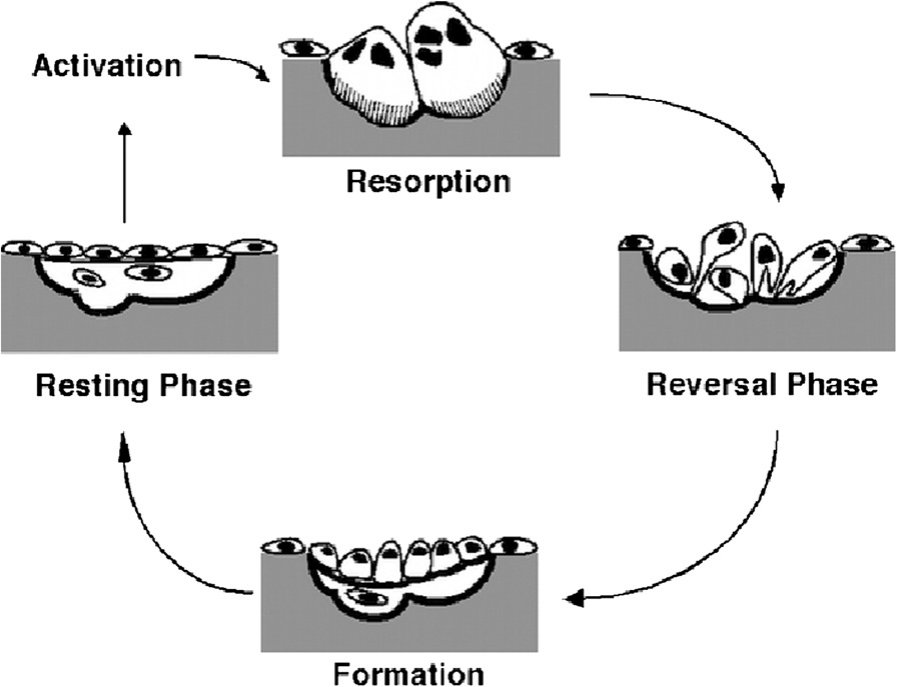
Bone remodeling sequence.

The bone remodeling process replaces approximately 20% of bone tissue annually; in healthy adults, bone remodeling occurs in a balanced, highly regulated manner in five phases: activation, resorption, reversal, formation, and quiescence as shown in Figure 1[3-5].

This process is assumed to repair the microdamage and maintain bone quality; and also occurs continuously with each cycle lasting 4 to 7 months [6].

Over the past, the progress made in understanding bone remodeling, through two groups: phenomenological approach and thermodynamic approach, has been truly spectacular.

The thermodynamic approach was initiated first by chemists and was applied to continuum mechanics by Eckart and Biot around 1950. Furthermore, this approach was adopted by introducing state variables [7,8] and thermodynamic potential which allows to define associated variables chosen for the study of the phenomenon [9].

Many theoretical and experimental works have been published emphasizing the importance of applying this thermodynamical approach to a bone structure [10-13].

In this study, we adopt thermodynamic approach of irreversible processes to get a new law of bone remodeling taking into account the bone density described by the law of Mullender et al. [14] and the damage evolution in the bone proposed by Martin [15].

The found equation is solved by the finite difference method (FDM) in the one-dimensional bone structure of a n-unit elements model.

Finally, we present some examples of numerical simulation results.

### Fundamental assumptions

In order to construct a general framework for the description of the bone remodeling process, the following simplified assumptions are made:

1) The bone is considered as a linear-elastic, isotropic and inhomogeneous material.

2) The external mechanical loading acts as a stimulus for bone remodeling.

3) The n-unit elements model is applied to the damaged-bone structure in the one-dimensional case.

4) The small perturbations hypothesis (displacements and their time and spatial variations are small).

5) The state coupling of damage with elastic strain.

6) The remodeling processes can be considered isothermal, adiabatic and without internal generation of heat.

7) The decoupling of the thermodynamic potential is assumed, such that:

$$\psi =\psi \left(\varepsilon^{e},D,\phi \right)=\psi_{e}\left(\varepsilon^{e},D\right)+\psi_{r}\left(\phi \right)$$

With:

*ψ*_*e*_(*ε*^*e*^, *D*) is the thermodynamic potential depending on the elastic strain tensor ε^*e*^ and the damage variable *D*.

*ψ*_*r*_(*ϕ*) is the thermodynamic potential depending on the bone density *ϕ*.

### Thermodynamic approach

The thermodynamics of irreversible processes allows the modeling of different materials behavior. This is accomplished by defining the state variables and the state potential and also the dissipation potential [16].

The general theory of adaptive damaged-elastic materials and general framework of continuum thermodynamics is considered to find a new law of bone remodeling [10,17].

A general definition of the thermodynamic forces associated with the internal variables is given by:

$$A_{k}=\rho \frac{\partial \psi }{\partial V_{k}}\;\left(k=1,2,\ldots \right)$$

Where conjugate forces *A*_*k*_ associated with internal variables (*V*_k_, k=1,2,…) by specification of the thermodynamic potential *ψ*(…, *V*_*k*_) as shown in Table 1[17,18].

**Table 1:** Thermodynamic variables

**State variables**		**Associated variables**
**Observable**	**Internal**	
*T*		*s*
*ε*		*σ*
	*V*_*k*_	A_*k*_

Within the hypothesis of small strains and small displacements, the state variables, observable and internal, are chosen in accordance with the physical mechanisms of deformation and degradation of the bone as follows [18].

Observable variables:

– *ε* is the total strain tensor associated with the stress tensor *σ*.

– *T* is the temperature associated with the specific entropy *s*.

Internal variables:

– *ε*^e^ is the elastic strain tensor associated with the stress tensor *σ*.

– *D* is the damage associated with a variable $\bar{\text{Y}}$.

– ϕ is the bone density associated with the bone remodeling variable *R*.

Table 2 summarizes the set of variables introduced [18].

**Table 2:** Chart of thermodynamic variables

**State variables**		**Associated variables**
**Observable**	**Internal**	
*T*		*s*
*ε*		*σ*
	*ε*^*e*^	*σ*
	*D*	$\bar{\text{Y}}$
	ϕ	- *R*

We assume a bone remodeling variable, which is characterized by:

*R* >*0* in the case of the formation phase.

*R* = *0* in the case of the equilibrium phase.

*R* <*0* in the case of the resorption phase.

We postulate the existence of a thermodynamic potential from which the state laws can be derived [16,18,19].

The state potential: *ψ* = *ψ*(*ε*^*e*^, *D*, *ϕ*)

We assume the following decoupling:

$$\psi =\psi_{e}\left(\varepsilon^{e},D\right)+\psi_{r}\left(\phi \right)$$

With:

*ψ*_*e*_(*ε*^*e*^, *D*) is the thermodynamic potential depending on the elastic strain tensor and the damage variable.

*ψ*_*r*_(ϕ) is the thermodynamic potential depending on the bone density.

The associated variables are defined by:

(1) $$\bar{\text{Y}}=\rho \frac{\partial \psi_{e}}{\partial D}$$

(2) $$-R=\rho \frac{\partial \psi_{r}}{\partial \phi }$$

The second law of thermodynamics imposes a restriction on dissipation which can be represented in terms of the Clausius-Duhem inequality [7,16,18] :

(3) $$\sigma :\overset{}{\varepsilon }-\rho .\overset{\cdot }{\psi }\geq 0$$

### The development of the Clausius-Duhem inequality

We note that:

$\rho \cdot \overset{\cdot }{\psi }=\rho \frac{\partial \psi_{e}}{\partial \varepsilon^{e}}:{\overset{\cdot }{\varepsilon }}^{e}+\rho \frac{\partial \psi_{e}}{\partial D}.\overset{\cdot }{D}+\rho \frac{\partial \psi_{r}}{\partial \phi }.\overset{\cdot }{\phi }$ with ε=ε^*e*^

and

(4) $$\sigma =\rho .\frac{\partial \psi_{e}}{\partial \varepsilon^{e}}$$

(the thermo-elasticity law) [17]

Inequality (3) may be written :

$$\sigma :\overset{\cdot }{\varepsilon }-\rho \frac{\partial \psi_{e}}{\partial \varepsilon^{e}}:{\overset{\cdot }{\varepsilon }}^{e}-\rho \frac{\partial \psi_{e}}{\partial D}.\overset{\cdot }{D}-\rho \frac{\partial \psi_{r}}{\partial \phi }.\overset{\cdot }{\phi }\geq 0$$

Using the previous equations (1) (2) (4), we obtain:

(5) $$-\bar{\text{Y}}.\overset{\cdot }{D}+R.\overset{\cdot }{\phi }\geq 0$$

## Discussion

In the case of constant damage: $\overset{\cdot }{D}=0$

The inequality (5) gives $+\text{R}.\overset{\cdot }{\phi }\geq 0$

In the resorption area:

***ϕ***˙ ≤0 and R<0

$$=>+\text{R}.\overset{\cdot }{\phi }\geq 0$$

In the formation area:

***ϕ***˙ ≥0 and R>0

$$=>+\text{R}.\overset{\cdot }{\phi }\geq 0$$

Equilibrium area (dead zone):

$\overset{\cdot }{\phi }=0\;\text{and}+\text{R}.\overset{\cdot }{\phi }=0$

$$=>+\text{R}.\overset{\cdot }{\phi }\geq 0$$

**Figure 2:**
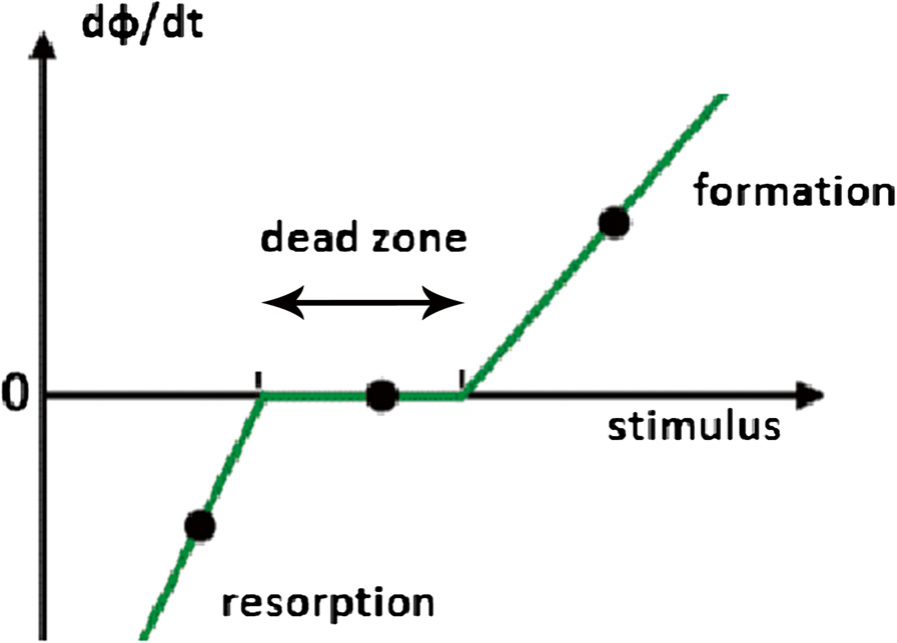
Law of bone remodeling.

We can conclude that the inequality (5) is verified in accordance with the law of bone remodeling as shown in Figure 2[20].

### The conservation of energy equation

The first principle of thermodynamics gives:

(6) $$\rho .\overset{\cdot }{e}=\sigma :{\overset{\cdot }{\varepsilon }}^{e}+r-div\;\vec{q}$$

[17,21]

With:

– e is the specific internal energy.

– r is the internal heat source.

– q is the heat flux.

We replaced *ρė* by the expression derived from *e* = *ψ* + *T*.*s*[17,21]

(7) $$\rho .\overset{\cdot }{e}=\rho .\overset{\cdot }{\psi }+\rho .\overset{\cdot }{T}.s+\rho .T.\overset{\cdot }{s}$$

and ρ *ψ* by its expression as a function of the state variables and the associated variables.

(8) $$\rho .\overset{\cdot }{\psi }=\sigma :{\overset{\cdot }{\varepsilon }}^{e}+\bar{\text{Y}}.\overset{\cdot }{D}-R.\overset{\cdot }{\phi }$$

We introduce the specific heat (capacity) defined by:

$\text{C}=\text{T}\frac{\partial \text{s}}{\partial \text{T}}$[16,17] and taking into account Fourier's law $\vec{q}=-k.g\vec{ra}dT$[17]

We obtain:

(9) $$div\;\vec{q}=-k.div\left(g\vec{ra}dT\right)=-k.\text{Δ}T$$

Using the previous equations (6) (7) (8) (9), we can get:

(10) $$\bar{\text{Y}}.\overset{\cdot }{D}-R.\overset{\cdot }{\phi }+\rho \overset{\cdot }{T}.s+\rho .T.\overset{\cdot }{s}=r+k.\text{Δ}T$$

### **Assumptions**

The classical heat equation corresponds to a process: [17]

Without internal generation of heat created by the external sources: *r*=0.

With adiabatic evolution: *k*. Δ*T* = 0.

With isothermal transformation: $\overset{\cdot }{T}=0$ Therefore $s=\frac{C}{T}.\overset{\cdot }{T}=0$ Then *s*=0.

Equation (10) may be written:

$$\bar{\text{Y}}.\overset{\cdot }{D}-R.\overset{\cdot }{\phi }=0$$

Then,

(11) $$R=\bar{Y}.\frac{\overset{\cdot }{D}}{\overset{\cdot }{\phi }}$$

We have: $\bar{\text{Y}}=-Y$

Where

(12) $$Y=\frac{1}{2}.E.{\overset{\cdot }{\varepsilon }}^{e}:{\overset{\cdot }{\varepsilon }}^{e}$$

(the strain energy release rate) [17]

We have also: $\rho .\psi_{e}=\frac{1}{2}.\left(1-D\right).E.{\overset{\cdot }{\varepsilon }}^{e}:{\overset{\cdot }{\varepsilon }}^{e}$ (the strain energy) [17] and the equivalent constraint σ_*eq*_ is written by $\sigma_{\mathrm{eq}}=\rho \frac{\partial \psi_{e}}{\partial \varepsilon^{e}}=\left(1-D\right).E.{\overset{\cdot }{\varepsilon }}^{e}$ (The thermo-elasticity law)

Then,

(13) $$\overset{\cdot }{\varepsilon }=\frac{\sigma_{\mathrm{eq}}}{\left(1-D\right).E}$$

With: *σ*_*eq*_ = *σ* in the one-dimensional case.

Using the previous equations (12) (13), equation (11) may be written:

(14) $$R=-\frac{\sigma^{2}}{2E.{\left(1-D\right)}^{2}}.\frac{\overset{\cdot }{D}}{\overset{\cdot }{\phi }}$$

The Young's modulus of the bone which is an isotropic material and inhomogeneous is expressed as:

*E* = (1 − *D*).*E*_0_ with *E*_0_ = *c*.*ϕ*^*α*^[22,23]

c=100 and α =3 are two constants characteristic of the bone

Then,

$$E=\left(1-D\right).c.\phi^{\alpha }$$

Equation (14) may be written:

$$R\left(\phi ,D\right)=-\frac{\sigma^{2}}{2c}.\frac{\overset{\cdot }{D}}{{\left(1-D\right)}^{3}}.\frac{1}{\phi^{\alpha }\overset{\cdot }{\phi }}$$

This equation represents the new law of bone remodeling developed by applying the thermodynamic approach in the context of damaged elastic.

In this study, we introduce the law of damage developed by Martin [15] which shows that the damage in human cortical bone can grow exponentially until the fatigue failure [15,24,25].

The evolution law for the damage is expressed as:

$D=D_{0}.e^{f_{d}t}$ Then, $\frac{\partial D}{\partial t}=\overset{\cdot }{D}=f_{d}D$

With:

*f*_*d*_ : the fatigue life of the bone devoid of the remodeling [26]

### *D*_0_ : the initial damage

*t* : the time

Finally, the new law of bone remodeling may be written as:

(15) $$R\left(\phi ,D\right)=-\frac{\sigma^{2}f_{d}}{2c}.\frac{D}{{\left(1-D\right)}^{3}}.\frac{1}{\phi^{\alpha }\overset{\cdot }{\phi }}$$

### Numerical resolution

The new law of bone remodeling (Equation 15) was solved numerically by dividing it into three parts:

1. a constant $\frac{\sigma^{2}f_{d}}{2c}$

2. a damage function $\frac{D}{{\left(1-D\right)}^{3}}$

3. a function of bone density $\frac{1}{\phi^{\alpha }\overset{\cdot }{\phi }}$

In this study, we use the law of bone density proposed by Mullender et al. [14]:

(16) $$\frac{\partial \phi_{i}}{\partial t}=\tau \overset{k=m}{\underset{k=1}{\sum }}e^{-\frac{d\left(i,I_{k}\right)}{d}}\left(\frac{S^{k}}{\phi_{k}^{\beta }}-S_{\mathrm{ref}}\right)$$

With :

– ϕ_*min*_ ≤ ϕ ≤ ϕ_*max*_

– ϕ_*min*_ is the density of completely resorbed bone

– ϕ_*max*_ is the maximum density defined for a compact bone

– *τ* is a positive constant related to the reaction time of bone tissue (constant of bone remodeling)

– 1≤ i ≤n

– ϕ_*i*_ density of bone tissue of element *i*

– *m* (*m* ≤ *n*) is the total number of osteocytes in the solid

– *I*_*k*_ (*1* ≤ *k* ≤*m*) corresponds to the series of numbers of the elements containing an osteocyte

– *S*_*k*_ represents the density of deformation energy in *I*_*k*_

– *S*_*ref*_ reference stimulus value

– *β* is a parameter reflecting the intensity of the stimulus cell

– *d* is the normalization factor limiting the area of influence of osteocyte

– *d*(*i*,*I*_*k*_) is the distance between the centers of geometric element *i* and the element *I*_*k*_

**Figure 3:**
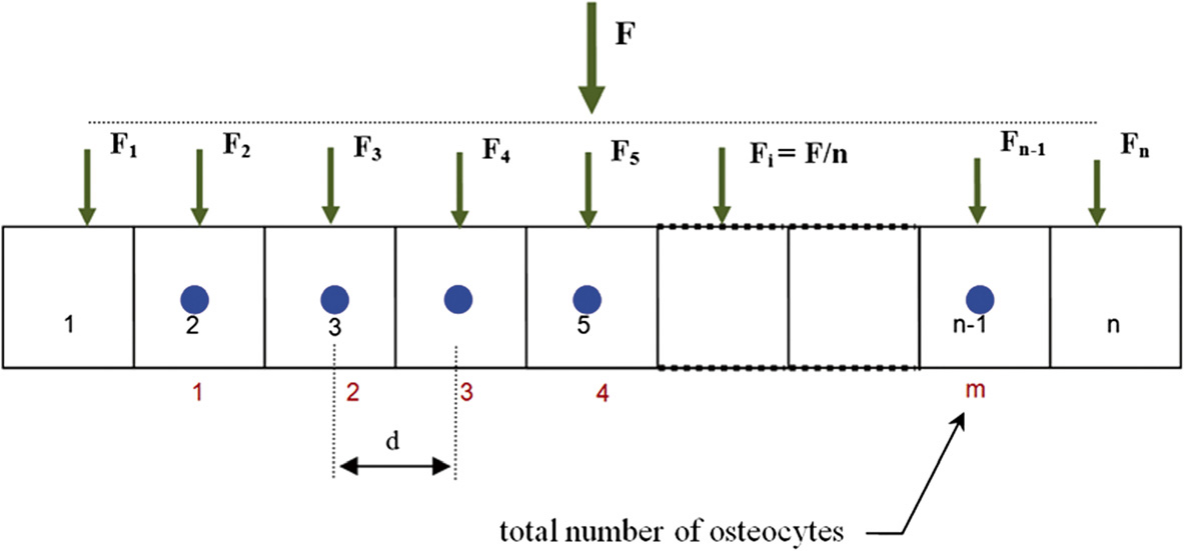
Model geometry with n-unit elements.

We discretize into n-unit elements a bone fragment and we apply a compressive force evenly distributed over the various units (Figure 3) [23,27].

To solve the previous equation (16), we use the method of finite difference with an implicit scheme and the fixed point method [23,27,28].

We obtain:

(17) $$\frac{\phi_{i}^{n+1}-\phi_{i}^{n}}{\text{Δ}t}=\tau \overset{k=m}{\underset{k=1}{\sum }}e^{-\frac{d\left(i,I_{k}\right)}{d}}\left(\frac{S^{k}}{\phi_{k}^{\beta ,n+1}}-S_{\mathrm{ref}}\right)\;i=1,2,n$$

$$\phi^{0}=\phi_{0}$$

## Results and discussion

We simulated the case of a uniform distribution of the osteocyte cells, and of another heterogeneous case. The values of the parameters used during the numerical simulations are given in Table 3[23,28].

**Table 3:** Values of the parameters used during the numerical simulations

**Data**	**Symbol**	**Values**	**Units**
Maximum density	ϕ_max_	1.75	g/cm^3^
Minimal density	ϕ_min_	0.01	g/cm^3^
Initial density	ϕ_0_	0.6	g/cm^3^
The step of time	Δt	5.10^-3^	UT
The total force	F	10	N
The distance between 2 centers	d	25	mm
Reference stimulus value	S_ref_	0.04	MPA
The fatigue life of the bone	f_d_	3	years
n-unit elements of the bone fragment		50	

m (m < n ) is the total number of osteocytes in the bone fragment.

Constants α = 3 β = 0.5 D_0_ = 0.8 c = 100 τ = 1.

**Figure 4:**
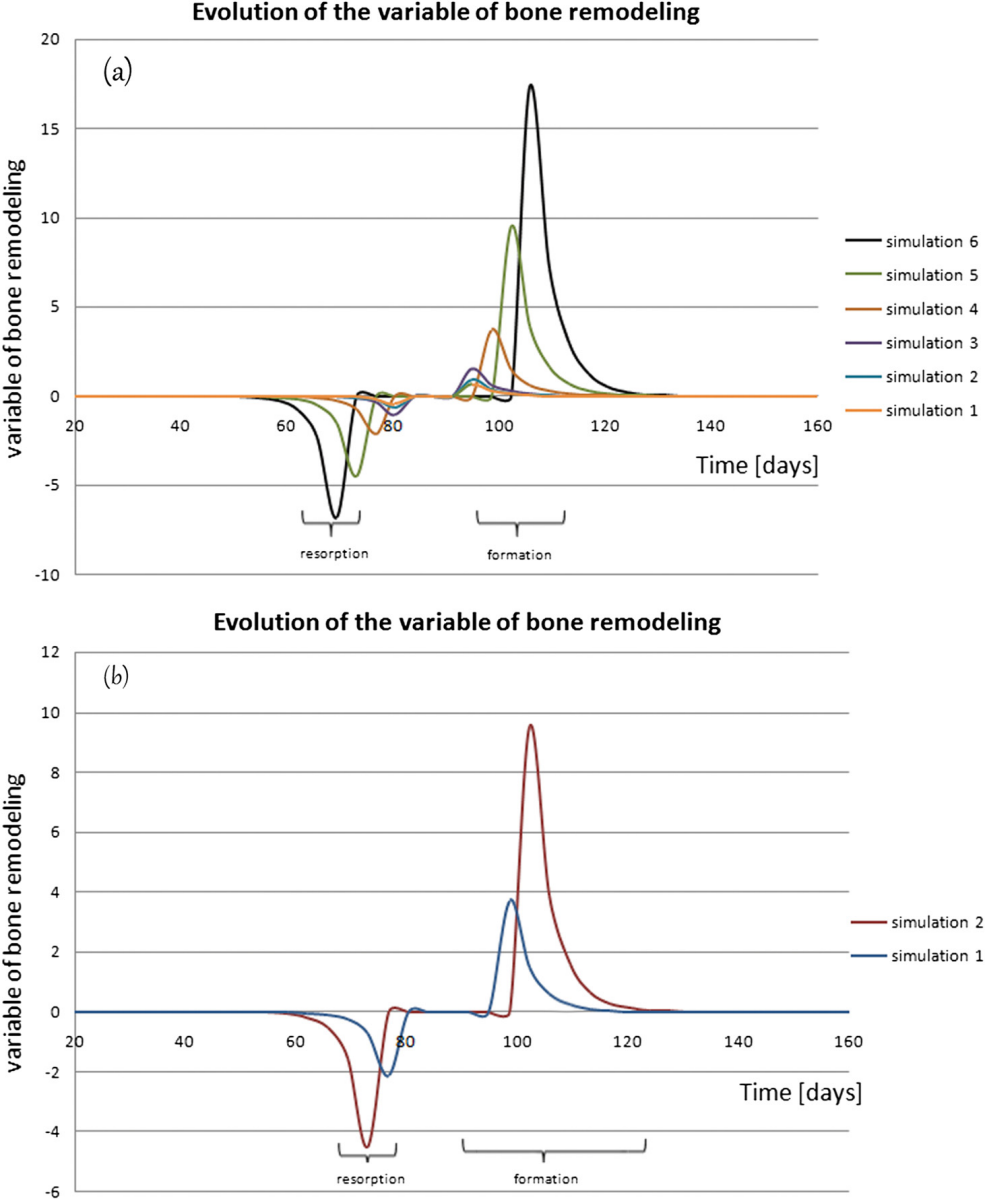
Evolution of the variable of bone remodeling, (a) case of a uniform distribution of the osteocyte cells, (b) a heterogeneous case.

Numerical results are shown in Figure 4.

Figure 4 shows the temporal evolution of the variable of bone remodeling in the case of a uniform distribution of the osteocyte cells (n=m=50), and of another heterogeneous case (n≠m with m=30 in the central package of the osteocytes).

The curves consists of three key periods. The first period of the curves corresponds to the resorption phase, where the variable of the bone remodeling was negative. The second period exhibits the formation phase, where the variable of the bone remodeling was positive. The third period defined as the interval between the resorption phase and the formation phase, which the variable of the bone remodeling reached zero.

The resorption phase takes approximately 18 days, which is then followed by an equilibrium phase that can last for up to 10 days and finally by the formation phase from 17 to 35 days. This is in agreement with results from the literature [6,29,30].

By comparing the curves in Figure 4 to the graph proposed by Terrier et al. [20], we see a good agreement. Furthermore, the curves that are found have a nonlinear shape.

## Conclusion

In this paper, we proposed a thermodynamic approach in small perturbations for bone remodeling process.

The adopted model takes into consideration both the bone density and the damage and gives a new law of bone remodeling. Then, the governing equation of the process was solved by the finite difference method in the one-dimensional bone structure with n-unit elements model.

The numerical results obtained are in accordance with the experimental results found in the literature.

## Competing interest

The authors declare that they have no competing interests.

## Authors’ contributions

Both authors contributed to writing and improving the paper and approved the final manuscript.

## Authors’ information

Ahmed Idhammad - He received his engineering degree status at the National School of Mineral Industry in Rabat, Morocco. He is curzently a Doctoral student at the Faculty of Sciences and Technics in Marrakech, Morocco. His research interests are numerical simulation, biomechanics, bone remodeling, thermodynamic, fatigue and damage.

Abdelmounaïm Abdali - PhD in Solid Mechanics and Structures in University of Amiens in 1996, France. He is a Professor in computer science at the University Cadi Ayyad, Faculty of Sciences and Technics, Marrakech, Morocco. Member at the Laboratory of Applied Mathematics and Computer Science (LAMAI) Marrakech, Morocco. His research interests are numerical simulation, biomechanics, bone remodeling and damage, computer science, DTN Network.
